# Mitogenic effect of sericin on mammalian cells

**DOI:** 10.1186/1753-6561-5-S8-P121

**Published:** 2011-11-22

**Authors:** Wataru Sato, Ken Fukumoto, Kana Yanagihara, Masahiro Sasaki, Yoshihiro Kunitomi, Satoshi Terada

**Affiliations:** 1 Department of Applied Chemistry and Biotechnology, University of Fukui, Fukui, 910-8507, Japan; 2 Development Department, SEIREN Co., Ltd., Fukui, 913-0038, Japan

## 

Fetal bovine serum (FBS) or other mammal-derived factors are extensively supplemented as growth factor into culture medium. Since mammal-derived factors arouses the concern about the risk of zoonosis, serum- and mammal-free culture is strongly required. We focused on sericin hydrolysates, originating from silkworm, and reported that sericin is effective as growth factor to various cells. But it is not elucidated how sericin induces the proliferation and inhibits apoptosis of the cells. In this study, inhibitor assay was done in order to identify signaling factors involved in sericin effect. In hybridoma cells, the involvement of *Src* was suggested, while *ERK1/2*, *PP2A* and *p38* were not involved. In HepG2 cells, the involvement of *Src* and *ERK1/2* was suggested. From these results, it was implied that the mitogenic effect of sericin might be transduced through different pathways depending on cell line.

## Background

In *in vitro* cell culture, mammal-derived factors including FBS are usually used as growth factors and supplemented into the media. However, supplementing mammal-derived factors causes the concern about the risk of zoonosis such as abnormal prions and various viruses. Therefore, mammal-free culture is strongly required in the industry of antibody therapeutics production and in regenerative medicine including cell therapy. As an alternative to mammal-derived factors, we focused on hydrolysate of sericin, which is glue protein included in silk fiber. Our previous studies revealed that sericin has mitogenic and anti-apoptotic effect on various cell lines [1-3]. Additionally, sericin hydrolysates treated with autoclave sterilization maintained its original mitogeneic activity and so we successfully developed a mammal-free medium, Sericin-GIT (Wako Pure Chemical Industries, Ltd., Japan). Although sericin is effective, it is not revealed how sericin up-regulates the proliferation and down-regulates apoptosis.

**Figure 1:**
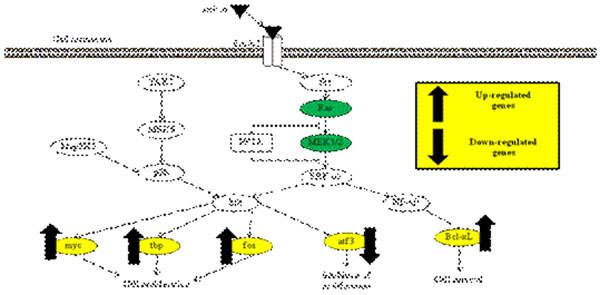
**The pathway deduced from our previous study.** cDNA microarray analysis detected up-regulation of *myc*, *tbp*, *fos*, and *Bcl-xL*, and down-regulation of *atf3* after sericin treatment. Inhibition assay confirmed the involvement of *Ras* and *MEK1/2* in the mitogenic effect of sericin.

In the previous study, the following results were obtained. One, cDNA microarray analysis detected up-regulation of *myc*, *tbp*, *fos* and *Bcl-xL*, and down-regulation of *atf3* after sericin treatment. Two, Inhibition assay confirmed the involvement of *Ras* and *MEK1/2* in the mitogenic effect of sericin. From these results, we suppose a following pathway (Figure 1). In this study, inhibitor assays against the factors shown in the figure were done in order to identify signaling factors involved in sericin effect.

## Materials and methods

**Cells.** Mouse hybridoma 2E3-O cells and human hepatoblastoma HepG2 cells were cultured in ASF104 (Ajinomoto Japan) serum-free medium and DME medium (Nissui, Japan) without FBS, respectively.

**Inhibitors**. PP2 (BioMoL, USA), ERK Activation Inhibitor 1, Cell-Permeable (Calbiochem, USA), Okadaic acid (Calbiochem), SB239063 (SIGMA, USA) were used at inhibition assay.

**Inhibition Assays.** Mouse hybridoma 2E3-O cells and human hepatoblastoma HepG2 cells were cultivated in addition of each of inhibitors. After two days, the number of cells and the viability were determined by trypan blue-exclusion test with hemocytometer.

## Results

Inhibition assays were done in order to identify signaling factors involved in sericin effect.

Since *Ras* and *MEK1/2* are involved in Map kinase pathway, PP2, a specific inhibitor against *Src*, was used. PP2 successfully neutralized the mitogenic effect of sericin, indicating that *Src* would be involved.

Involvement of *ERK1/2* was tested by using ERK Activation Inhibitor 1. The inhibitor neutralized the mitogenic effect in HepG2 cells, but did not in hybridoma cells, suggesting that *ERK1/2* would be involved in HepG2 cells but not in hybridoma cells. In order to confirm that *ERK1/2* is not involved in hybridoma cells, inhibition assay against *PP2A* was done; the mitogenic effect could be enhanced by inhibition of *PP2A* if MAPK pathway would be involved in the mitogenic effect of sericin. Inhibition against *PP2A* failed to improve the proliferation of the hybridoma cells treated with sericin, implying that *PP2A* would not be involved.

Further cDNA microarray analysis implied that several other genes might be affected by sericin treatment. Among the genes, *myc* and *stat1* are the lower signaling factors of *p38* and so SB239063, a specific inhibitor against *p38*, was tested. It failed to neutralize, indicating that *p38* would not be involved in the mitogenic effect of sericin.

## Conclusions

**Figure 2:**
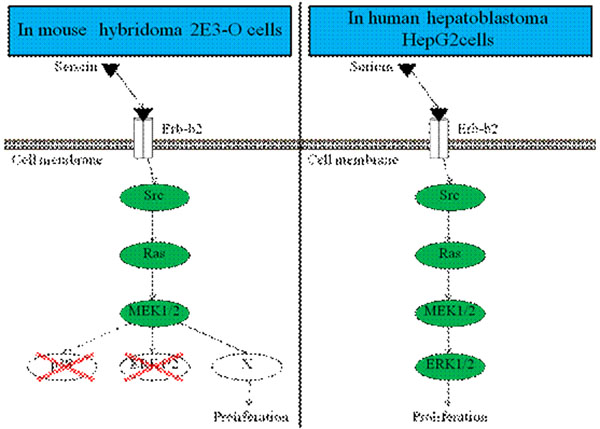
Different pathways depending on cell line.

In hybridoma cells, *Src* was involved in the mitogenic effect of sericin, while *ERK1/2*, *PP2A* and *p38* were not, suggesting that *Src* could activate other factors to transduce signal of sericin. In HepG2 cells, the involvement of *Src* and *ERK1/2* was confirmed, suggesting that MAPK pathway could be involved. From these results, the mitogenic effect of sericin is transduced through different pathways depending on cell lines as shown in figure 2. Further study should be done to reveal the involvement of these factors.
